# Dexamethasone as an additive to bupivacaine in an ultrasound-guided adductor canal block for the management of persistent pain after arthroscopic reconstruction of the anterior cruciate ligament: a randomized, double-blind study

**DOI:** 10.1186/s12871-025-02921-6

**Published:** 2025-04-24

**Authors:** Saeid Elsawy, Amani Abdelwahab, Yara Hamdi, Rasha Ahmed Ali Hamed

**Affiliations:** 1 Anesthesia and Intensive Care Department, Faculty of Medicine, Assiut University, Asyut, Egypt; 2 Anesthesia and Intensive Care Department, Assiut University, Asyut, Egypt

**Keywords:** PPSP, ACL reconstruction, Adductor canal block

## Abstract

**Background:**

Pain is a protective response to noxious stimuli to prevent further damage. The surgical incision results in several events that lead to pain that outlasts its benefits. Persistent postsurgical pain (PPSP) is defined as “pain that persists for three months after surgical intervention.

**Objectives:**

To investigate the effect of dexamethasone as an adjuvant to adductor canal block on persistent postsurgical pain after ACL reconstruction.

**Patients and methods:**

Ninety patients who underwent arthroscopic reconstruction of the anterior cruciate ligament and who completed the study were randomly allocated into two groups. Forty-five patients in each group received ultrasound-guided adductor canal block at the end of surgery. G I (Dexa group): Forty-five patients received a 20 ml mixture of 0.25% bupivacaine and 8 mg dexamethasone (2 ml). G II (control group): Patients received a 20 ml mixture of 0.25% bupivacaine and 2 ml of normal saline.

**Results:**

No significant differences in demographic data, intraoperative hemodynamics or surgery duration were detected between the two groups. The duration of postoperative analgesia was significantly longer in the dexamethasone group (10 h ± 3 vs. 6 h ± 1) than in the control group. Additionally, 24 h postoperative consumption of meperidine was significantly lower in the dexamethasone group (65 ± 23 vs. 104 ± 27) than in the control group. Postoperative VAS scores at 6 and 12 h were also lower in the dexamethasone group than in the control group. Three-month follow-up revealed a significantly lower incidence of PPSP in the dexamethasone group (20% versus 33% in the control group). Moreover, the severity of PPSP was significantly lower in the dexamethasone group than in the control group. Additionally, Codeine consumption was significantly lower in the dexamethasone group than in the control group.

**Conclusion:**

Perineural dexamethasone in ultrasound-guided adductor canal block reduced the severity of PPSP and opioid analgesia consumption in the first three months following arthroscopic reconstruction of the ACL.

**Clinical trial registration:**

The study was registered on clinical trial registration (NCT04631822) in October 2020.

**Supplementary Information:**

The online version contains supplementary material available at 10.1186/s12871-025-02921-6.

## Introduction

Persistent postsurgical pain (PPSP) is defined as “pain that persists for three months after surgical intervention; that was not present before intervention. The characteristics of PPSP differ from those of preoperative pain, which is localized to the surgical site with the exclusion of other causes of pain (e.g., malignancy recurrence, infection). Chronic postsurgical pain (CPSP) can lead to limitations in activities and psychological burdens, as well as disappointment and frustrating feelings of the surgical team. Therefore, preventive measures to minimize PPSP should be considered [1]. Although arthroscopic reconstruction of the anterior cruciate ligament is a common minimally invasive ambulatory knee surgery, it is associated with moderate to severe postoperative pain [2]. Persistent pain after ACL reconstruction can be severe enough to limit daily and sports activities, with an incidence ranging from 5–22%, but the cause remains uncertain [3–5]. The major risk factors for PPSP are the type of surgery, surgical technique, and perioperative pain level. The level of preoperative pain is one of the strongest and most consistent independent risk factors for PPP regardless of surgery type [6–8]. Specifically, for ACL reconstruction, regional analgesia (RA) has become an essential component of the postoperative multimodal analgesic regimen [8]. RA not only improves acute postoperative pain but also attenuates chronic postoperative pain [9, 10]. Compared with other orthopedic medical procedures, including rotator cuff repair, meniscal debridement, and labral repair, ACL reconstruction has subjectively been ranked as the most painful [11].

Dexamethasone is the most commonly used regional anesthesia adjuvant for prolonging the postoperative analgesic effect of peripheral nerve block, with satisfying results [12–14]. To the best of our knowledge, no trials have investigated the effect of perineural dexamethasone on persistent postoperative pain after ACL reconstruction. We hypothesize that dexamethasone might be able to decrease incidence of persistent postoperative pain due to its anti-inflammatory properties and its ability to prevent C fiber mediated nociception and block ectopic neuronal firing. The aim of the present study investigated the effect of dexamethasone, an adjuvant to local anesthetics in ultrasound-guided adductor canal blocks, on persistent postsurgical pain after ACL reconstruction.

## Patients and methods

**Fig. 1:**
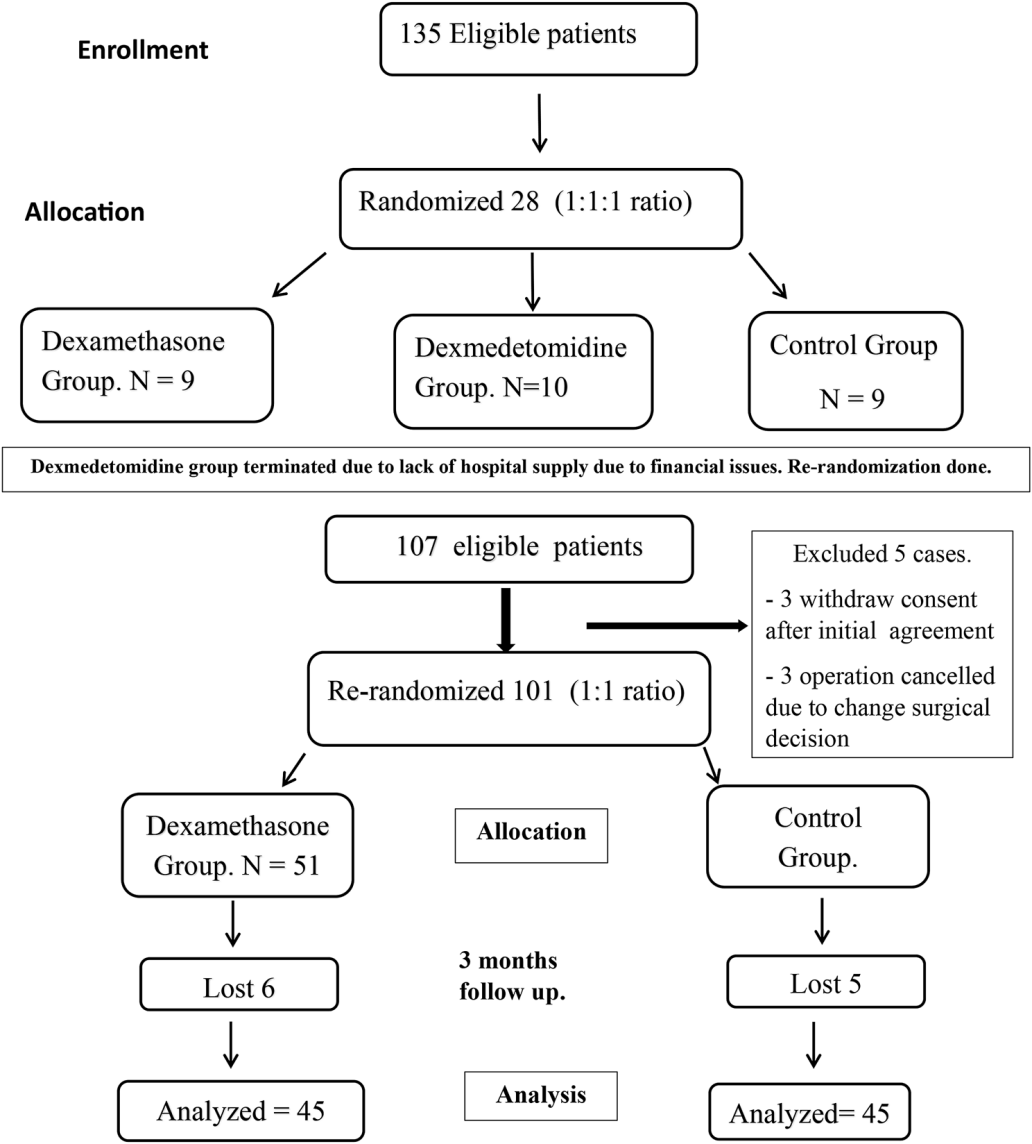
Consort flow chart

This randomized, double-blinded controlled trial was conducted at Assiut University Hospital after ethical approval from our local committee (No IRB300364), the study was registered on clinical trial registration (NCT04631822) in October 2020. This trial follows CONSORT guidelines and the Declaration of Helsinki. The study was designed to compare the effects of the addition of dexmedetomidine and dexamethasone to bupivacaine on pain control after ACL reconstruction. However, due to a lack of dexmedetomidine supply after starting the study, this group was terminated, and rerandomization was performed at a 1:1 ratio (Fig. 1). Ninety patients, from January 2021 to December 2023, completed the study. Patients were randomly allocated using computer generated random blocks of 10 at 1:1 ratio into two groups:

G I (dexamethasone group): Forty-five patients received a 20 ml mixture of 0.25% bupivacaine and 8 mg dexamethasone (2 ml) injected into the adductor canal under ultrasound guidance at the end of surgery.

G II (control group): Forty-five patients received 20 ml of a mixture of 0.25% bupivacaine and 2 ml of normal saline injected at the adductor canal under ultrasound guidance at the end of surgery. The group assignments were kept in a sealed opaque envelope that opened on the morning of surgery at our local research pharmacy. The drug mixture was prepared by a pharmacist under septic conditions in opaque vials labeled by patient number and research medication. Patients, the data collector and the anesthetist responsible for the block were blinded to the nature of the research mixture. Adult patients aged between 19 and 60 years of both sex and American Society of Anesthesiologists (ASA) physical status I or II were included in the study after providing written informed consent. Patients who refused to consent or withdraw after initial consent, were ASA III or IV, experienced continuous preexisting pain, had known contraindications to peripheral nerve block, including local skin infections, bleeding diathesis, and coagulopathy, were excluded from the study.

The primary outcome was the dynamic visual analog score (DVAS) 1 month after ACL reconstruction. The secondary outcomes included the postoperative VAS score at 24 h, DVAS score at 2 months and 3 months, time to first analgesic request, and opioid consumption during the first 24 h postoperative and subsequent three months postoperative.

### Intervention

ACL reconstruction was performed under spinal anesthesia. At the end of surgery, sterilization of the thigh was performed. A high-frequency (L6-12HZ) linear ultrasound transducer (GE machine- logic F6 model-Italy) was placed transverse to the longitudinal axis at the mid-thigh level, at a distance halfway between the iliac spine and the patella. The femoral artery was identified underneath the sartorius muscle with the vein just underneath the artery. At this position, the saphenous nerve was placed lateral to the artery in the adductor canal. A single shot nerve block echogenic needle was inserted in plane from the lateral side of the transducer through the sartorius muscle with the tip placed lateral to the artery. Twenty milliliters of 0.25% bupivacaine mixed with either dexamethasone or saline was injected to expand the canal.

### Measures

In the first 24 h postoperative the measured data were the mean VAS score at 2, 6, 12, and 24 h, time to first analgesic request and total opioid consumption. In the next three months after surgery, both the DVAS and the total amount of codeine consumed per day were evaluated at the first, the second and the third month postoperative.

The rescue analgesia in the first 24 h was paracetamol 1 gm if the VAS score was less than 4 and tramadol 50 mg if the VAS score was more than 4. The regimen for postoperative pain ,*at home after hospital discharge*, was Solpadine, Smithkline Beecham S.A. Spain, (500 mg paracetamol + 8 mg codeine + 50 caffeine) 4 to 6 times daily as needed, with gradual withdrawal starting from the 7th postoperative day and replacement with 400 mg ibuprofen after telephone consultation with the pain physician responsible for data collection. Postoperative visits to the pain clinic were scheduled for participants at 1, 2 and 3 months postoperative. The telephone number of all participants was taken, and if they skipped the scheduled visit, they were telephoned by the data collector.

### Sample size

According to a previous study [15], the mean VAS score for persistent pain after ACL reconstruction was 1.6 at the 6th week after surgery, and the standard deviation (SD) was ± 1.1. A total of eighty patients (40 patients in each group) were needed to detect a 50% reduction in the VAS score between the groups 1 month after surgery, with 90% power and an alpha error of 0.05. To compensate for dropouts, we included ninety patients.

### Statistical analysis

IBM SPSS software version 22.0 (SPSS Inc., Chicago, IL) was used. The data are presented as the means ± SDs, medians (ranges) and numbers (%) as appropriate. Independent t tests were used to compare means between parametric data. Mann‒Whitney tests were used to compare nonparametric values in the studied groups, and repeated measures analysis will be used for repeated data (blood pressure, heart rate, and visual analog scale (VAS) scores and codeine consumption). The chi-square test or Fisher’s exact test will be used for categorical data. A P value > 0.05 was considered statistically significant.

## Results

**Fig. 2:**
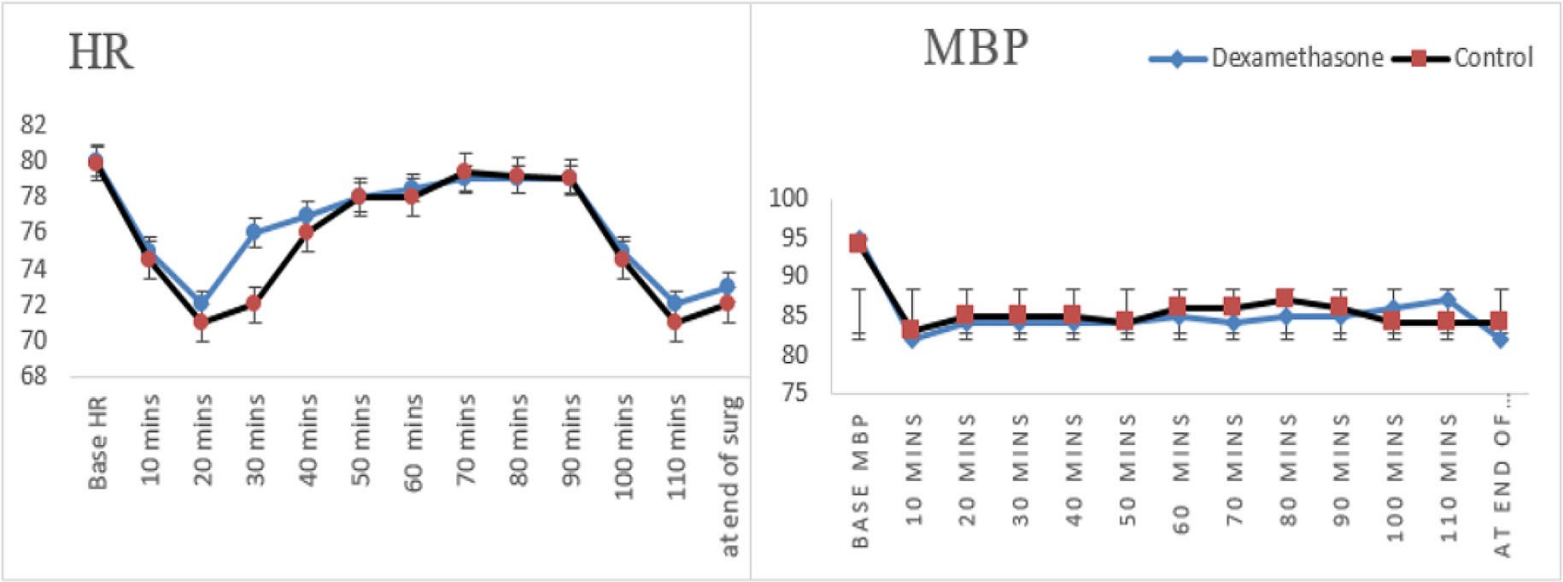
Intraoperative hemodynamics

Ninety patients out of 107 completed the study (Fig. 1). Patients were randomly allocated at a 1:1 ratio into two groups: G I (Dexa group): patients received a 20 ml mixture of 0.25% bupivacaine and 8 mg dexamethasone (2 ml) injected at the adductor canal under ultrasound guidance at the end of surgery. G II (control group): Patients received a 20 ml mixture of 0.25% bupivacaine and 2 ml of normal saline injected at the adductor canal under ultrasound guidance at the end of surgery. No statistically significant differences were found in the demographic data, surgery duration or ASA status between the study groups (Table 1). No significant differences were found in the baseline or intraoperative hemodynamics [mean blood pressure (MBP) and heart rate (HR)] between the studied groups (Fig. 2). The analgesia time, represented by the time to first analgesic request by the patient, was significantly longer in the dexamethasone group than in the control group (10 h ± 3 vs. 6 h ± 1 with *P* < 0.001, respectively) (Table 1). Additionally, the total amount of tramadol consumed as rescue analgesia in the first 24 h after surgery was significantly lower in the dexamethasone group than in the control group (65 ± 23 vs. 104 ± 27; *P* < 0.001, respectively) (Table 1).

**Table 1:** Demographic data, ASA status, surgery duration and postoperative data

	Dexamethasone group	Control group	*P* value
	(*n* = 45)	(*n* = 45)	
Age (years)	44 ± 8	42 ± 10	0.07
CI	(41–64)	(39–45)	
Sex			
Male	24 (53%)	25 (56%)	0.3
Female	21 (47%)	20 (44%)	
Body mass index (kg/m^2^)	27 ± 3	26 ± 2	0.5
CI	(25–27)	(25-26.5)	
ASA class			
Class-I	39 (87%)	40 (89%)	0.2
Class-II	6 (13%)	5 (11%)	
Surgery duration (min)	107 ± 9	106 ± 8	0.9
CI	(105–107)	(104–109)	
First analgesic request (hour)	10 ± 3	6 ± 1	< 0.001
CI	(9–11)	(5.7–6.3)	
24 h tramadol consumption (mg/day)	65 ± 23	104 ± 27	< 0.001
CI	(59–73)	(96–113)	
No of patients suffering from pain after 3 months postoperative	9 (20%)	16 (35.5%)	
ARR	15.50%		
NNT	6.5		

Data are expressed as frequencies (percentages) or means± (SDs). Chi-square for categorical data, t-test for parametric data. The P value was considered significant if it was < 0.05. **ASA**: American Society of Anesthesiologists. BMI: body mass index. CI; confidence interval

**Fig. 3:**
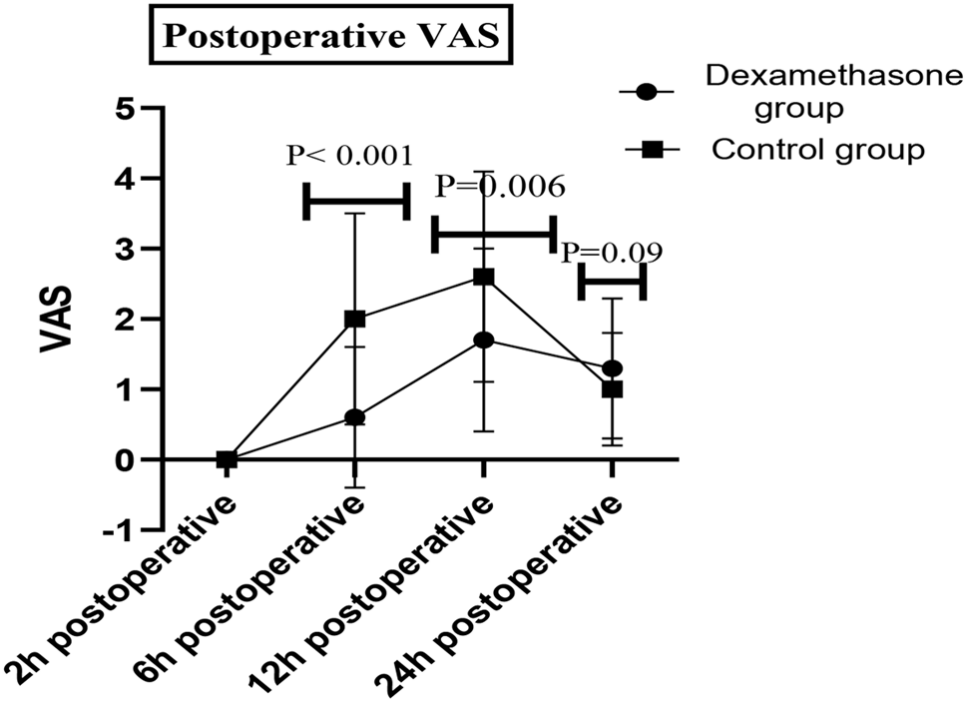
Postoperative VAS score

**Fig. 4:**
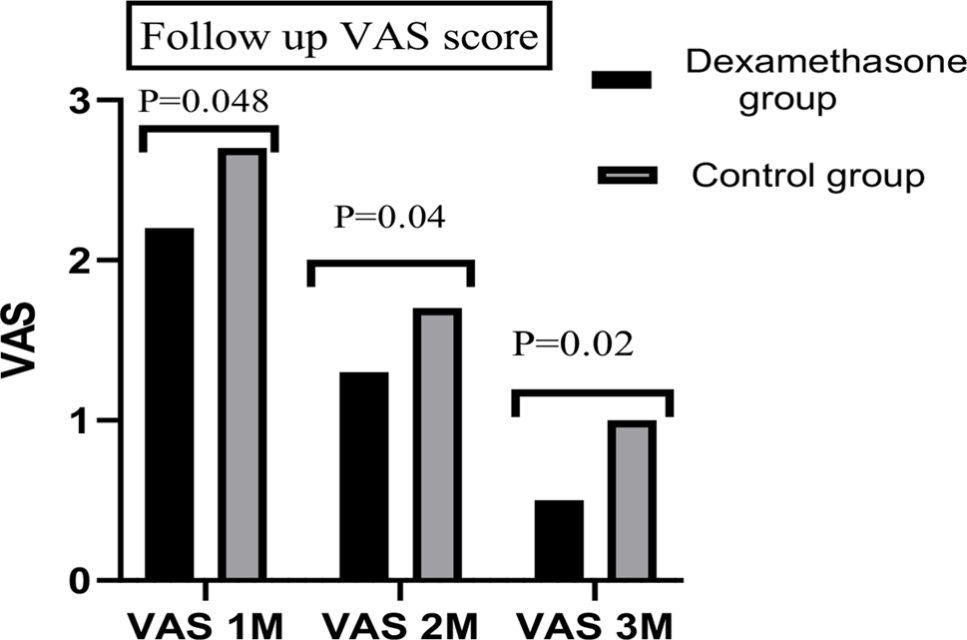
Three-month VAS score follow-up

**Fig. 5:**
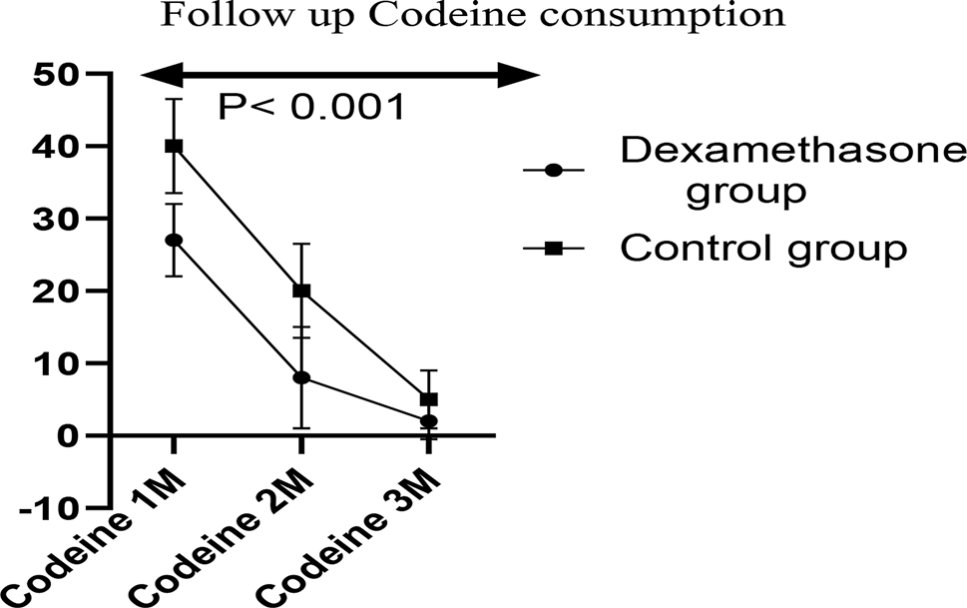
Three-month postoperative Codeine consumption

The VAS score was significantly lower in the dexamethasone group than in the control group at 6 h and 12 h (*P* < 0.001 and 0.006, respectively). However, no significant difference was found in the VAS score at 24 h postoperative (*P* = 0.09; Fig. 3; Table 2). Three-month follow-up revealed significantly lower VAS scores in the dexamethasone group than in the control group at the first, second and third months after surgery (Fig. 4), with an absolute risk reduction (ARR of 15.5%) and a number needed to treat (NNT) of 6.5 (Table 1) . Moreover, codeine consumption in the first, second and third months after surgery was significantly lower in the dexamethasone group than in the control group, with highly significant time*group interactions; *P* < 0.001, F = 31 and df = 1.7 (Fig. 5).

**Table 2:** Comparison of 24-h postoperative VAS scores between the study groups

Time of assessment	Dexamethasone group (*n* = 45)	Control	*P* value
		group (*n* = 45)	
2 h-postoperative	0 ± 0	0 ± 0	0
6 h-postoperative	0.6 ± 1	2 ± 1.5	< 0.001
CI	(0.4–0.9)	(2–3)	
12 h-postoperative	1.7 ± 1.3	2 ± 1.6	0.006
CI	(1-2.1)	(1.5–2.5)	
24 h-postoperative	1.3 ± 1	1 ± 0.8	0.09

The data are expressed as the means (SDs). Mann-Whitney test for group comparison. The *P* value was considered significant if it was < 0.01

## Discussion

Persistent postsurgical pain is a chronic pain syndrome that results from surgical procedures. Acute postoperative pain ideally resolves within 1–2 weeks with pain control regimens. Unfortunately, in some patients, acute postoperative pain is sustained beyond normal time limits for tissue healing and progresses into a “chronic” or persistent postoperative pain state. Persistent postsurgical pain (PPP) is defined as pain that lasts for > 3 months and is confined to the surgical field or to the nerve supply of the surgical field or to a dermatome after surgery in deep somatic or visceral tissue [5].

Postoperative pain is a highly specific entity of combined inflammatory and neuropathic pain. In animal studies, an incisional pain model demonstrated cellular and molecular changes that differ from those in other pain models [16]. Furthermore, surgical injury can induce alterations in gene expression in the cell bodies of peripheral nociceptors (within the dorsal root ganglion), which results in prolonged excitability and nociceptive firing, leading to central sensitization and the transition from acute to chronic pain [17]. Molecular and cellular studies on surgical incision-induced pain have shown that the maintenance of hyperalgesia does not require continuous exposure of nociceptors to inflammatory mediators that initiate hyperalgesia through the c-AMP pathway; rather, it is maintained by increased expression of protein kinase A (PKA), a key gene in neuropathic pain, in spinal cord neurons [18, 19]. PKA activates the tetrodotoxin-resistant sodium channel that is primarily found in nociceptors and is thought to regulate the inflammatory mediator-induced sensitization of nociceptors [18]. Regional anesthesia is anticipated to reduce the incidence of PPSP via many mechanisms; blocking the transmission of pain and inflammation from nociceptors to the CNS and the anti-inflammatory properties of local anesthetics themselves could limit neuronal inflammation and the activation of glial cells. In addition, regional anesthesia is acknowledged for minimizing intraoperative and postoperative opioid consumption, thus reducing the possibility of opioid-induced hyperalgesia [16]. Furthermore, the use of additives could attenuate pain and inflammation. Dexamethasone, despite its non-FAD approval, is a famous local anesthetic additive that has a beneficial role in prolonging the analgesic effect of local anesthetics [16]. The results of the present study agreed with our hypothesis that the beneficial effect of perineural dexamethasone might extend beyond the amelioration of acute postoperative pain and ameliorate persistent postsurgical pain after arthroscopic ACL reconstruction. VAS scores at the 3-month follow-up were significantly lower in the dexamethasone group. Moreover, the amount of codeine consumed was also reduced in the dexamethasone group. Dexamethasone is usually used to treat neural inflammation following nerve injury. In a rat model, local injection of dexamethasone in a transected sciatic nerve enhanced the repair of the sciatic nerve and its target organ reinnervation. This effect is explained by the local anti-inflammatory and potential neurotrophic effects of dexamethasone at the site of nerve injury. This leads to reduced inflammatory cell infiltration and inflammatory mediator production at the site of nerve injury [20]. Moreover, dexamethasone has an antineuropathic effect on pain. It enhances the phosphorylation of PKA and increases the spinal cord microglial expression of dynorphins (endogenous opioids) through the upregulation of dynorphin A, which inhibits pain transmission to supraspinal centers [21].

The present study is limited by its sample size and unicentric and short-term follow-up. PPSP is a complex syndrome that requires more research and long-term follow-up.

## Conclusion

perineural dexamethasone in ultrasound-guided adductor canal block reduced the severity and incidence of PPSP and opioid consumption in the first three months following arthroscopic reconstruction of the ACL.

## Electronic supplementary material

Below is the link to the electronic supplementary material.

Supplementary Material 1

## Data Availability

The datasets used and analyzed during the current study are available from the corresponding author upon reasonable request.
